# Rationale and design of the British Heart Foundation (BHF) Coronary Microvascular Angina (CorMicA) stratified medicine clinical trial^☆^^☆☆^

**DOI:** 10.1016/j.ahj.2018.03.010

**Published:** 2018-07

**Authors:** Thomas J. Ford, David Corcoran, Keith G. Oldroyd, Margaret McEntegart, Paul Rocchiccioli, Stuart Watkins, Katriona Brooksbank, Sandosh Padmanabhan, Naveed Sattar, Andrew Briggs, Alex McConnachie, Rhian Touyz, Colin Berry

**Affiliations:** a West of Scotland Heart and Lung Centre, Golden Jubilee National Hospital, Glasgow, United Kingdom; b British Heart Foundation Glasgow Cardiovascular Research Centre, Institute of Cardiovascular and Medical Sciences, University of Glasgow, Glasgow, United Kingdom; c University of New South Wales, Sydney, Australia; d Centre for Population and Health Sciences, University of Glasgow, Glasgow, United Kingdom; e Robertson Centre for Biostatistics, Institute of Health and Wellbeing, University of Glasgow, Glasgow, United Kingdom

## Abstract

**Background:**

Coronary angiography is performed to assess for obstructive coronary artery disease (CAD), but “nonobstructive CAD” is a common finding. Microvascular or vasospastic angina may be relevant, but routine confirmatory testing is not evidence based and thus rarely performed.

**Aim:**

The aim was to assess the effect of stratified medicine guided by coronary function testing on the diagnosis, treatment, and well-being of patients with angina and nonobstructive CAD.

**Design:**

The BHF CorMicA trial is a prospective, multicenter, randomized, blinded, sham-controlled trial of stratified medicine (NCT03193294). All-comers referred for elective coronary angiography for investigation of suspected CAD will be screened. Following informed consent, eligible patients with angina and nonobstructive CAD will be randomized 1:1 immediately in the catheter laboratory to either coronary artery function–guided diagnosis and treatment (intervention group) or not (control group). Coronary function will be assessed using a pressure-temperature–sensitive guidewire and adenosine followed by pharmacological testing with intracoronary acetylcholine. Patients will be stratified into endotypes with linked therapy. The primary outcome is change in Seattle Angina Questionnaire score at 6 months. Secondary outcomes include safety, feasibility, diagnostic utility (impact on diagnosis and diagnostic certainty), and clinical utility (impact on treatment and investigations). Health status is a key secondary outcome assessed according to the following domains: quality of life, treatment satisfaction, illness perception, physical activity, and anxiety-depression score. Patients with obstructive disease who are not randomized will form a registry group who will be followed up as a comparator for secondary outcomes including health status. Health and economic outcomes will be evaluated in the longer term using electronic health record linkage.

**Value:**

CorMicA is a proof-of-concept clinical trial of a disruptive stratified intervention with potential benefits to patients and health care providers.

## Graphical abstract

**Figure:**
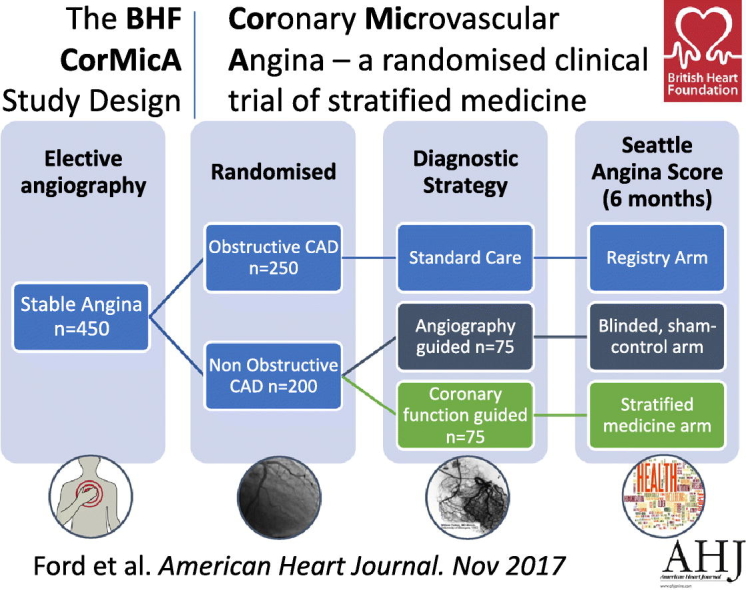

## Background

### Stable coronary syndromes: looking beyond epicardial coronary artery disease

Typically, the diagnosis of angina focuses on detecting obstructive epicardial coronary artery disease (CAD). This allows evidence-based medical treatment including myocardial revascularization in patients with obstructive CAD. Recent clinical trials, including ORBITA^1^ and the CIAO substudy of ISCHEMIA (NCT02347215), have stimulated pause for thought about the causes of angina beyond obstructive CAD.

Approximately 4 in 10 patients with angina undergoing elective coronary angiography have nonobstructive CAD.^2^ Between 2004 and 2008 in the United States, among almost 400,000 patients undergoing coronary angiography, 39.2% had no evidence of epicardial CAD.^2^ In addition, angina often persists following revascularization procedures.^3^ The reasons for a “negative” coronary angiogram are multifactorial, although many of these patients may have a disorder of coronary artery function including microvascular or vasospastic angina.4., 5. The term *stable coronary syndrome* has been proposed to increase physician awareness of these important diseases.6., 7. They may both cause ischemia with nonobstructive coronary artery disease.^8^ A recent working group called for research to improve the diagnosis and management of this common clinical problem.^9^

**Figure 1:**
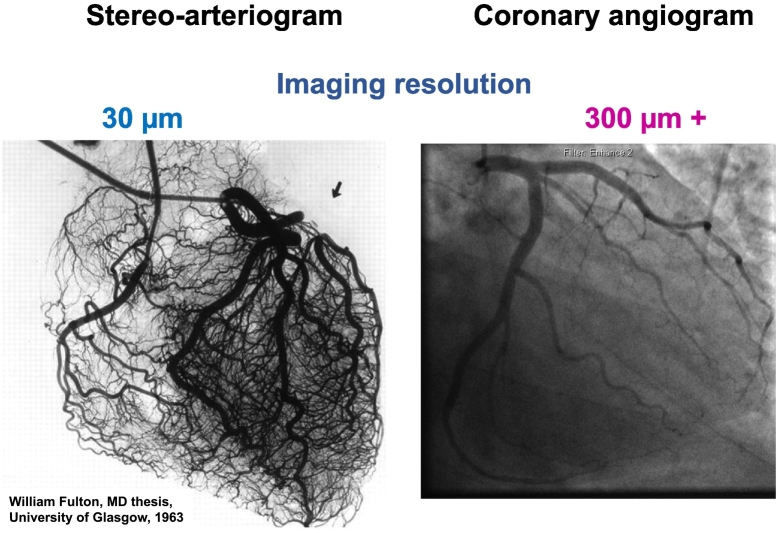
**Diagnostic coronary angiography lacks sensitivity to detect coronary microvascular dysfunction.** The postmortem stereoarteriogram (left), obtained by infusion of a bismuth microsolution at physiological levels of blood pressure, reveals angiographically smooth coronary arteries with no evidence of CAD. The arteriogram reveals microvessels and innate collateral connections. Image reproduced with permission: William Fulton, MD Thesis (1963), University of Glasgow. The angiogram (right) of the left coronary tree is normal with no evidence of CAD. The microvessels are invisible on the standard angiogram. These images highlight that diagnostic coronary angiography lacks sensitivity in detecting coronary microvascular dysfunction. Clinicians lack information about microvascular and/or vasospastic angina that may lead to suboptimal treatment.

The coronary microcirculation includes a trabecular network of arterioles and microvessels (≤400 μm), which are the final pathways for delivery of blood to the heart.^10^ Visual assessment of the coronary angiogram may identify coronary blood vessels of approximately 0.5-mm diameter; however, smaller vessels are not visualized (Figure 1).

The term *coronary microvascular dysfunction* describes functional and/or structural changes in the microcirculation that may result in microvascular angina (MVA). Without objective coronary artery function testing, the diagnosis and onward treatment for patients with chest pain and a “negative” angiogram are varied. Importantly, patients with undiagnosed chest pain (including those who have undergone cardiac investigations) are at increased risk of cardiovascular events in the longer term.^11^

### Coronary vasomotor, diagnostic tests, and stratified medicine

Coronary flow reserve (CFR) reflects the vasodilator capacity of the epicardial artery and its microcirculation^12^ and is prognostically important.^13^ CFR may be selectively measured within a coronary artery using a Doppler or a pressure-temperature–sensitive guidewire with thermodilution technique. The index of microvascular resistance (IMR) is a parameter that quantitatively reflects microvascular resistance in a straightforward way using a diagnostic guidewire as an adjunct to invasive angiography.^14^ These metrics provide complementary information on coronary artery and microvascular function.^15^ Pharmacological testing with intracoronary infusion of acetylcholine (ACh) and glyceryl trinitrate provides information on endothelial-dependent and endothelial-independent function, respectively. A pharmacological protocol involving infusion of incremental concentrations of ACh provides information on the propensity to vasospasm of the microvessels or epicardial coronary artery (as revealed by invasive coronary angiography). Stratified medicine is the identification of key subgroups of patients (endotypes) within a heterogeneous population, these endotypes being distinguishable by distinct mechanisms of disease and/or responses to therapy.^16^ We propose a stratified approach to angina involving comprehensive diagnostic tests to define endotypes that align with evidence-based therapy.

### ANOCA: evidence of endotypes linked to treatment strategies

In patients with angina and nonobstructive CAD (ANOCA), the stratifier is adjunctive use of coronary function tests during coronary angiography to rule in or rule out microvascular angina, vasospastic angina, both, or neither (normal coronary function). The study by Tremmel’s group further confirmed the feasibility of invasive physiological testing in a reasonably large, selected population. They noted distinct subgroups of patients with over 75% of the cohort having an occult explanation for their angina.^17^

*Microvascular angina* is a heterogeneous clinical syndrome, and its pathogenesis is incompletely understood. In CorMicA, we align this endotype with the COVADIS working group definition of MVA.^18^ This broad group encompasses various types of coronary microvascular dysfunction in patients with ANOCA. Our protocol for this endotype (Table II) is consistent with MVA guidelines from the European Society of Cardiology based on symptom relief and therapy for underlying coronary microvascular dysfunction.^8^ Our protocol is first-line symptomatic relief of MVA with β-blockers. Calcium channel blockers (CCBs) are recommended where β-blockers are not tolerated or ineffective. Nicorandil or ranolazine can be added; however, nitrates are avoided in MVA without vasospasm owing to potentially deleterious effects on exercise tolerance and lack of efficacy.19., 20. Where blood pressure and patient preference permit, statins and angiotensin-converting enzyme inhibitors (ACEIs)^21^ are recommended as potentially disease -modifying agents targeting underlying coronary microvascular dysfunction.

*Vasospastic angina* (VSA) is prognostically important and underdiagnosed despite having effective treatment options.^22^ In CorMicA, CCBs are first-line therapy with symptomatic and prognostic benefit.^23^ β-Blockers which are used first line in MVA should be avoided because they may precipitate spasm.^24^ Although nitrates are avoided for those with predominant MVA, they often provide symptomatic benefit for patients with VSA. Smoking cessation and use of statins are encouraged for pleiotropic benefits on endothelial dysfunction.

*Noncardiac chest pain* with fully normal coronary artery function testing is an important diagnosis for both patients and physicians (Table II). Undiagnosed chest pain is predictive of major adverse cardiac events,^11^ whereas quality of life suffers in patients recently diagnosed with ischemic heart disease.^25^ Stopping inappropriate antianginal therapy and secondary preventative medications may provide health and economic benefits to the patient and health care system.

### Rationale: standard care of patients with angina and a “negative” coronary angiogram

Diagnostic tests of coronary vasomotion are rarely used in clinical practice. Crucially, there is a lack of evidence from randomized, controlled clinical trials that treatment linked to results of coronary function tests improves patient well-being and delivers economic value. Consequently, there are no practice guideline recommendations. Furthermore, clinicians may lack training and experience in the use of these tests. The pathophysiology may be uncertain, and without evidence, the additional time and cost cannot be justified. This gap underpins potential suboptimal management and outcomes.

We will address these gaps through a randomized, controlled clinical trial of stratified medicine. We will include mechanistic studies to better understand the pathophysiology of endotypes to develop novel therapies.

## Overall objective

The objectives were to assess the effect of stratified medicine guided by coronary function testing on the diagnosis, treatment, and well-being of patients with ANOCA on invasive coronary angiography. The stratification involves use of complementary tests of coronary function to diagnose endotypes (subgroups) with protocol linked therapy and guidance to support clinicians’ ongoing management.

Disorders of coronary artery function, including coronary microvascular dysfunction and vasospasm, are common in patients with ANOCA as revealed by coronary angiography.26., 27. These disorders are prognostically important28., 29. and associated with high symptom burden, health care resource utilization, and long-term risk of major adverse cardiac events.11., 28., 30., 31.

## Hypothesis

Our hypothesis is that routine adjunctive testing of coronary function will facilitate diagnosis of endotypes and patient stratification. This personalized approach, including pharmacological and nonpharmacological interventions that are aligned to the patient’s endotype, will lead to improvement in angina and well-being compared to standard care without knowledge of coronary function.

## Study design

**Figure 2:**
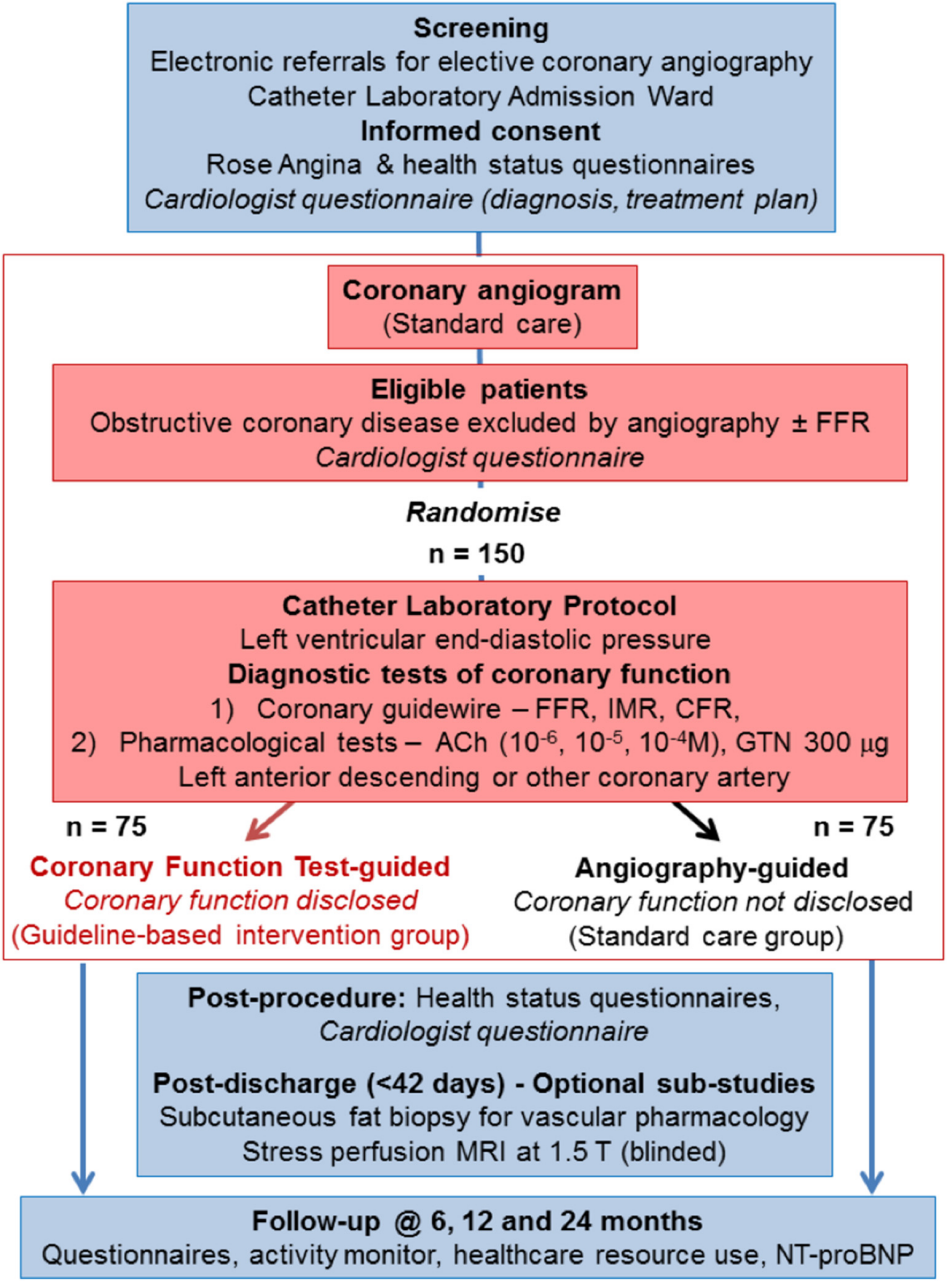
Schematic study design: flow diagram.

The British Heart Foundation (BHF) CorMicA trial is a prospective, double-blind, randomized, sham-controlled clinical trial comparing 2 management approaches to the clinical problem of patients with stable angina without obstructive coronary disease on invasive angiography. In the intervention group, a stratified medicine approach will be implemented involving diagnosis of endotypes with linked therapy. The control group involves current optimal management based on standard coronary angiography (Figure 2).

### Setting

The study takes place in 2 large regional hospitals (Golden Jubilee National Hospital and Hairmyres Hospital) providing invasive care to all patients in the West of Scotland (population 2.5 million).

### Eligibility criteria

Consecutive outpatients undergoing clinically indicated elective diagnostic angiography for investigation of suspected angina (typical or atypical angina according to Rose angina questionnaire^32^) are screened and invited to participate (Figure 2). Potential participants have angina medication withheld (except for sublingual nitroglycerin and aspirin) for 24 hours prior to coronary angiography. Informed consent is obtained before the coronary angiogram. Exclusion criteria (Table I) include a noncoronary indication for angiography (eg, valvular heart disease or hypertrophic cardiomyopathy), significant renal impairment (estimated glomerular filtration rate <30 mL/min) and obstructive disease on angiography (ie, >50% lesion by diameter stenosis in epicardial artery >2.5mm or a fractional flow reserve [FFR] ≤0.80). A minimum of 400 consecutive patients undergoing elective invasive coronary angiography patients is expected to be screened to enroll 150 subjects within 24 months. Consenting patients who are not randomized, for example, because of the presence of obstructive disease or for logistical reasons, will enter into a registry involving similar follow-up to the randomized trial participants, including completion of health status questionnaires and follow-up >3 years using electronic case record linkage.

**Table I:** Eligibility criteria

Inclusion criteria
Aged ≥18 y
A clinically indicated plan for invasive coronary angiography
Symptoms of angina (according to the Rose and Seattle angina questionnaires).

Exclusion criteria
A noncoronary indication for invasive angiography, eg, valve disease, hypertrophic obstructive cardiomyopathy
During the angiogram: obstructive disease evident in a main coronary artery (diameter >2.5 mm), ie, a coronary stenosis >50% or an FFR ≤0.80.⁎
Substudies: contraindication to contrast-enhanced CMR, eg, severe renal dysfunction (glomerular filtration rate <30 mL/min), non–CMR-compatible pacemaker or defibrillator.

*CMR*, cardiovascular magnetic resonance.

⁎ Patients with obstructive disease are eligible to participate in the registry follow-up.

### Randomization, implementation, and blinding

Participants are enrolled by research staff on the ward before the angiogram is performed. The standard care management strategy is established and recorded before randomization. The treatment plan is based on all of the clinical information including the results of the angiogram. FFR may be measured in intermediate lesions of uncertain functional significance. If the eligibility criteria are fulfilled, the patient will then be randomized in the catheter laboratory to the intervention group (coronary function tests disclosed) or standard care group with invasive angiography only (coronary function tests measured but not disclosed) using a Web-based randomization system provided by the Robertson Centre for Biostatistics (University of Glasgow). The randomization sequence involves block lengths randomized in blocks of length 4, that is, every 20 allocations consists of 4 blocks, 2 of length 4 and 2 of length 6, in a random order. The allocation sequence is on a 1:1 basis between the intervention group and the control group. The sequence is concealed electronically. Patients are considered to be randomized as soon as the allocation is assigned on the Web-based portal.

### Blinding and adherence

#### Angiography-guided group and blinding

In patients randomized to the angiography-guided group, coronary function tests are measured in the same way as in the intervention group except that the results will not be disclosed. The research staff obscure the hemodynamic monitor from the clinicians, nurses, and patients such that it is impossible for them to observe the guidewire information either in the catheter laboratory or afterward. Electronic displays on other hemodynamic monitors that may have been visible in the catheter laboratory are disabled. Complete blinding is ensured with the use of a second cardiologist (Dr Tom Ford) to supervise intracoronary ACh while monitoring the angiogram for visual display of coronary diameter and electrocardiogram during which time the attending clinician is blinded. Quality control checks, including assessments of equalized pressure recordings and verification of symptoms and hemodynamic changes with intravenous adenosine, are conducted in the usual way. Pharmacological tests are performed in an identical fashion in both groups. Adherence to monitoring is prospectively recorded by the research staff.

### Diagnostic strategy groups

#### Disclosed: coronary artery function testing group

Invasive coronary artery function are measured and disclosed after the coronary angiogram, permitting the cardiologist to identify endotypes, reclassify the initial diagnosis based on coronary angiography, and personalize therapy, including pharmacological and lifestyle measures informed by practice guidelines (Table II and Supplementary File 1).^8^

**Table II:** Definitions of coronary artery function disorders with linked therapy

Endotype	Disorder of coronary artery function		Linked pharmacotherapy
Microvascular angina (nonobstructive CAD and proven CMD)	↑ Microvascular resistance	IMR ≥25. IMR is a quantitative method for specifically assessing microvascular function independent resting hemodynamics. IMR = distal coronary pressure * transit time (average time for 3 saline bolus runs at hyperemia).	Baseline therapy: Consider aspirin, statin and ACEI therapy in all patients. PRN sublingual GTN Antianginal Rx 1st Line: β-blocker (eg, carvedilol 6.25 mg BD uptitrated) 2nd Line: CCBs substituted (non-DHP, eg, verapamil 40 mg BD uptitrated) where β-blockers are not tolerated or ineffective. 3rd line: add in therapy • CCB: DHP (eg, amlodipine)—only for those on β-blockers • Nicorandil (5 mg BD, uptitrated) • Ranolazine (375 mg BD, uptitrated)
	↓ Coronary vasorelaxation	CFR by thermodilution <2.0. This reflects the inability to increase coronary flow above 2 times the resting flow.	
	↓ Microvasodilator capacity	Resistive reserve ratio <2.0. This reflects the vasodilator capacity of the microcirculation to change from baseline to hyperemia (resistance at rest divided by resistance at hyperemia).	
	Microvascular spasm	Angina during ACh infusion or bolus with typical ischemic ST-segment changes and epicardial coronary constriction <90% reduction in epicardial coronary artery diameter. Represents inappropriate susceptibility microvascular constriction.	
Vasospastic angina	Epicardial spasm	*Epicardial coronary artery spasm* is defined as a reduction in coronary diameter >90% following intracoronary ACh in comparison with baseline resting condition following intracoronary GTN administration in any epicardial coronary artery segment together with symptoms and ST segment deviation on the electrocardiogram.	Baseline therapy: If atherosclerosis or endothelial impairment, aspirin and statin should be considered. PRN sublingual GTN Antianginal Rx 1st line: CCB, eg, verapamil 40 mg BD uptitrated 2nd line: add nitrate, eg, isosorbide mononitrate 10 mg BD 3rd line: change nitrate to nicorandil, eg, nicorandil 5 mg BD
Mixed MVA/VSA	CMD and epicardial vasospasm	Epicardial spasm plus any abnormality of •Microvascular resistance •Coronary vasorelaxation •Microvasodilator capacity	Baseline therapy: Consider aspirin, statin, and ACEI therapy in all patients. PRN sublingual GTN Antianginal Rx 1st line: CCB, eg, verapamil 40 mg BD uptitrated 2nd line: add nicorandil 5mg BD
Obstructive CAD		>50% lesion by diameter stenosis in epicardial artery >2.5 mm or an FFR ≤0.80^30^	Baseline therapy: If atherosclerosis or endothelial impairment, patients should be considered for aspirin, statin, and ACEI therapy Consideration of revascularization, antianginal therapy as per ESC guidelines.
Noncardiac	Nil	Exclusion of diffuse or obstructive epicardial coronary disease (FFR >0.8) without any of the following abnormalities of coronary function: CFR <2.0, IMR ≥25, or functional angina/spasm during ACh.	Cessation of antianginal therapy. stop antiplatelet and statin unless other indication Consider noncardiac investigation or referral where appropriate (eg, psychology, gastroenterology)

*ACh*, Acetylecholine; *CMD*, coronary microvascular dysfunciton; *VSA*, vasospastic angina; *GTN*, glyceryl trinitrate; *ESC*, European Society of Cardiology.

#### Blinded: usual care group

Invasive coronary artery function is measured but not disclosed; management is based on standard coronary angiography alone.

### Protocol: coronary physiology measurements

CFR, IMR, and FFR are measured using previously described methods.14., 33. In brief, intravenous heparin (50-70 U/kg) will be administered, and a guiding catheter without side holes is used to interrogate the coronary artery. A pressure-temperature sensor guidewire (PressureWire X, Abbott Vascular, Santa Clara, CA) wirelessly transmits data to a personal computer with dedicated analysis software (Coroventis, Uppsala, Sweden). The wire sensor tip is positioned at the tip of the guiding catheter, and the pressure measurement from the wire is equalized with that of the guiding catheter. The sensor is then positioned in the distal third of the coronary artery followed by 3 intracoronary injections of saline (3 mL) at room temperature. The mean transit time is measured with each bolus and averaged to calculate the resting mean transit time. An intravenous infusion of adenosine (140 μg·kg^−1^·min^−1^) will be administered via a large peripheral or central vein to induce steady-state maximal hyperemia, and 3 more injections of 3 mL of room temperature saline will then be performed. The transit time is automatically measured after each set of injections and averaged to calculate the hyperemic mean transit time. Simultaneous measurements of mean aortic pressure (by guiding catheter) and mean distal coronary pressure (by pressure wire) are also made during maximal hyperemia. IMR is calculated as the distal coronary pressure at maximal hyperemia multiplied by the hyperemic mean transit time.^14^ Increased IMR (≥25) is representative of microvascular dysfunction.^17^ CFR is calculated using thermodilution as resting mean transit time divided by hyperemic mean transit time^34^ (*abnormal CFR* is defined as ≤2).^35^ FFR is calculated by the ratio of mean distal coronary pressure to mean aortic pressure at maximal hyperemia; *abnormal FFR* is defined as ≤0.80.^33^

### Coronary vasoreactivity testing

The target coronary artery is the left anterior descending coronary artery. If technical factors, for example vessel tortuosity, preclude assessment of this artery, then the left circumflex or right coronary artery will be selected. Assessment of endothelium-dependent coronary vasomotor function will be performed by intracoronary infusion of ACh via the guiding catheter at concentrations of 0.182, 1.82, and 18.2 μg/mL (10^−6^, 10^−5^, and 10^−4^ mol/L, respectively) infused at 1 mL/min for 2 minutes via a mechanical pump.^36^ An assessment of symptoms and a 12-lead electrocardiogram are performed before starting the infusion and then again after each infusion period. The coronary angiogram is also performed at each time point in an identical projection that delineates the artery without foreshortening. A final test of the propensity to coronary vasospasm will be performed using 100 μg of ACh (5.5 mL of 10^−4^ mol/L over 20 seconds). This is reduced to 50 μg of ACh if the right coronary artery is interrogated. Epicardial artery spasm may be focal or diffuse. Focal constriction is defined as a circumscribed transient vessel narrowing within the borders of 1 isolated or 2 neighboring coronary segments. Diffuse constriction is diagnosed when the vessel narrowing is observed in ≥2 adjacent coronary segments.^37^ Finally, coronary angiography is repeated following an intracoronary bolus of 300 μg (3 mL) of nitroglycerin (Abbott Laboratories, Santa Clara, CA), an endothelium-independent vasodilator.

### Quantitative coronary angiography

Quantitative coronary analysis of the target artery, for example, left anterior descending, will be performed using computer-assisted angiographic analysis (QAngio XA7.3, Medis, Leiden, Netherlands) by trained cardiologists at the Golden Jubilee National Hospital Core Laboratory who are blinded to the clinical and physiological results. The coronary artery (typically left anterior descending artery) measurements are performed in the region where the greatest change had occurred during coronary reactivity testing.^38^ End-diastolic cine frames that best show the segment are selected, and calibration of the video and cine images is accomplished with the diameter of the guide.

The definitions of coronary artery function disorders are shown in Table II. Patients with obstructive disease are eligible to participate in the registry follow-up. Coronary artery diameter change (% from baseline) will be measured in response to both ACh and glyceryl trinitrate. *Severe endothelial dysfunction* is defined by ≥20% luminal constriction during ACh infusion (up to 10^−4^M); this finding implies significant reduction in coronary artery blood flow with prognostic implications when compared with patients whose arteries are <20% constricted.^39^

## Outcomes

### Primary outcome

Health status and symptoms will be assessed at baseline and again at 6 months using the Seattle Angina Questionnaire (SAQ).^40^ The primary end point is the mean difference in the within-subject change in SAQ between the groups at 6 months from baseline. A team of health professionals blinded to group allocation will send and collate the questionnaires from study participants. The SAQ is the most widely used and validated patient-reported outcome measure for angina. Although derived from a mostly male cohort with obstructive CAD, validation studies in women have been completed showing prognostic value.41., 42., 43. In the WISE and iPOWER cohorts, SAQ severity correlated with physiologic metrics of coronary microvascular dysfunction.44., 45. It has been used extensively in other studies of ANOCA.25., 46., 47. Furthermore, in the landmark COURAGE study, the SAQ proved a valid metric of functional angina and quality of life in patients despite apparently successful PCI.^48^

### Secondary outcomes

The key prespecified secondary outcomes are described in Table III. The independent clinician responsible for the patients’ care adjudicates on the final diagnosis which is prospectively completed on a predischarge questionnaire (Supplementary File 2). Where patients have both MVA and a positive ACh test result for epicardial vasospasm, the treating physician will document the main diagnosis based on their interpretation of results in the context of the clinical presentation. Reclassification of diagnosis after disclosure of detailed coronary artery function results will be assessed. Participant contacts will continue for up to 3 years, and then longer-term follow-up for medication use and health outcomes will continue using electronic record linkage. A key feature of the study design is the inclusion of obstructive patients in a registry who are followed up for health status and adverse events.

**Table III:** Secondary outcomes

Secondary outcomes		Time frame
Feasibility and safety	Feasibility of the stratified medicine approach defined by protocol compliance as measured by deviations from the protocol.	Up to 3 y
	Enrolment rates, procedure duration, and protocol compliance relating to enrolment, crossover, integrity of blinding, adherence with therapy during follow-up, and compliance with follow-up assessments.	
	Procedure-related serious adverse events	Day 1
Diagnostic utility	Diagnosis of endotypes (disease strata): obstructive CAD, coronary vasospastic angina, microvascular angina, endothelial dysfunction (no angina), normal (noncardiac, normal coronary function results, no angina).	Day 1
	Impact of disclosure of the coronary function test results on the diagnosis and certainty of the diagnosis (diagnostic utility)	
Clinical utility	Impact of disclosure of the coronary function test results on medical decisions (including treatment and investigations) and to compare these decisions against medical decisions formed by an independent panel of experts (reference data set).	Day 1
	Assess the relationships between cardiovascular risk factors, reflected by validated risk scores (eg, ASSIGN, JBS3), and parameters of coronary function in medically managed patients.	
Vascular function	To assess whether patients have abnormal peripheral vascular function (using in vitro wire myography studies of vascular function).	42 d
Cardiac stress perfusion MRI	Assess the diagnostic accuracy of stress perfusion magnetic resonance (CMR) imaging for identification of endotypes based on reference tests of coronary function.	42 d
	Detection of clinically significant (actionable) incidental findings using magnetic resonance imaging. The incidental findings may be cardiac or noncardiac.	
	Detection of myocardial pathology using multiparametric CMR	
Health status	Health status and symptoms will be assessed at baseline and again at 6 m, 12 m, and closeout using the SAQ. The secondary outcome is the within-subject change in SAQ score over time.	6, 12, and 24 m and/or closeout up to 3 y
	Assess the participants' general health status and self-reported quality of life using the EQ5D questionnaire.	
	Assess the participants' self-reported levels of anxiety and depression using the Patient Health Questionnaire-4	
	Assess the participants' self-reported levels of treatment satisfaction using the Treatment Satisfaction Questionnaire for Medication	
	Assess the participants' perception of their illness using the brief Illness Perception Questionnaire	
	Assess the participants' self-reported activity during daily life (Duke Activity Status Index) and physical activity (International Physical Activity Questionnaire)	
Obstructive disease registry	Health status and symptoms will be assessed using the SAQ and health status questionnaires for patients with obstructive disease entered into the registry. A comparison of mean within-subject change in the health status domains above will be made between the registry patients and the randomized study groups.	6 m
Health economics	Assess resource utilization including primary and secondary care costs for tests, procedures and outpatient visits, and medicines between the randomized groups	Up to 36 m

ASSIGN, ASSIGN score estimates a person's risk of developing cardiovascular disease developed for use in Scotland; JBS3, Joint British Societies recommendations on the prevention of Cardiovascular Disease (*JBS3*) is a calculator for cardiovascular disease risk.

### Feasibility

The study has been peer-reviewed by the British Heart Foundation, and the protocol has been approved by the West of Scotland Research Ethics Service (Reference 16/WS/0192). The trial will be conducted in line with *Guidelines for Good Clinical Practice in Clinical Trials*.

## Statistical considerations

The CorMicA trial has a comprehensive statistical analysis plan that governs all statistical aspects of the study authored by the trial statistician before any unblinded data are seen. The analysis plan is based on intention-to-treat principles in line with CONSORT guidelines. It focuses on estimation of treatment effect differences with 95% CIs and *P* values. All prespecified secondary outcome analyses will be reported in study publications to further inform efficacy, safety, and cost-effectiveness. Continuous outcomes will be analyzed using linear regression with adjustment for baseline levels where these are available. Where continuous data are clearly not normally distributed (eg, laboratory variables), standard transformations will be applied to achieve approximate normality prior to analysis. Appropriate alternative regression methods will be applied to other types of data (eg, logistic regression for binary outcomes). The angiographic parameters will be correlated with clinical outcomes. Clinical data will be made available to the data coordinating center (the Robertson Centre for Biostatistics) through an electronic clinical report form.

### Sample size calculation

To detect a mean group difference of change in SAQ score of 9 units, we calculated that a sample size of 70 patients per group gave 80% power to detect a between-group difference in SAQ score between the groups. This calculation assumed the 5% significance level (2-sample *t* test of the mean group difference of within-subject ΔSAQ). This projected effect is consistent with other studies, for example, the observed difference in the change in SAQ frequency score with ranolazine (9.4; *P* = .027) versus placebo in patients with a reduced CFR.^45^

### Follow-up and timetable

Quality of life and health status questionnaires (Table III) will be completed at 6, 12, and 24 months (or study close out, whichever comes sooner). Follow-up assessments for adverse events will be performed by the clinical research staff by telephone or in person (eg, outpatient clinic review), as appropriate. Medical records will also be checked. Follow-up contact will occur at 6-month intervals until the last patient has achieved a minimum of 6-month follow-up. Follow-up in the longer term (ie, ≥3 years) will be supported by electronic record linkage with central government health records. The active phase of the project will be completed within 30 months. Follow-up procedures will be the same for patients in both of the groups. Written management guidance for each endotype, informed by practice guidelines,^8^ will be provided to the cardiologists, general practitioners, and nurse practitioners with advice to start, stop, and optimize treatment (including nonpharmacological/lifestyle measures) in line with the final diagnosis (Supplementary File 1).

### Ancillary studies

Laboratory analyses will focus on mechanistic pathways causally implicated in abnormalities of coronary artery function and myocardial perfusion. These will include endothelial cytokines, markers of inflammation (eg, high-sensitivity C-reactive protein), metabolic status (plasma glucose, lipids), and biomarkers of cardiovascular dysfunction (eg, N-terminal pro b-type natriuretic peptide (NT-proBNP)). These will be obtained at baseline and repeated at 12 months. Multiparametric stress perfusion cardiac magnetic resonance imaging at 1.5 T (Magnetom Avanto, Siemens Healthcare) will be obtained within 42 days of enrolment. Incidental findings will be disclosed, but the stress perfusion results will be blinded. All patients will be offered the opportunity to participate in a substudy of peripheral vascular biology undertaken using wire myography of peripheral resistance vessels isolated from gluteal subcutaneous fat biopsies.

### Trial management and governance

Standard operating procedures will be used, and adverse events will be adjudicated by clinicians independent of the research group and blind to the study data. Progress in the trial will be monitored by the Trial Manager (Katriona Brooksbank, PhD) and Sponsor representatives from Pharmacy and Research Management. The study will be subject to internal and external audit that is routinely coordinated by the Sponsor. Based on the safety experience in a recent diagnostic study^17^ in which no serious adverse events were reported, a Data and Safety Monitoring Committee will be constituted should any serious adverse event arise. An annual report will be submitted to the Ethics Committee on a 12-month basis. The flow diagram illustrates conservative estimates of patient enrolment and activity on a single site. The study will follow STARD (http://www.equator-network.org/reporting-guidelines/stard/) and CONSORT (http://www.consort-statement.org/) guidelines. Adverse events are defined and reviewed as outlined in Supplementary File 3.

### Ethics

The BHF CorMicA trial has full UK National Research Ethics Service approval (Reference 16/WS/0192).

### Registration

The ClinicalTrials.gov registration is NCT03193294.

### Sources of funding

CorMicA is an investigator-initiated clinical trial that is funded by the British Heart Foundation (PG/17/2532884; RE/13/5/30177). No companies are involved in this study.

The trial sponsor is the Golden Jubilee Research Foundation. The authors are solely responsible for the design and conduct of the study, all analyses, the drafting and editing of the paper, and its final contents.

## Discussion

Diagnostic tests of coronary function are rarely performed for patients with ANOCA in daily practice. To date, these tests have mainly been used for research purposes. On the other hand, the tests may be considered as “reference” measures for the diagnosis of disorders of coronary function. There are several reasons why coronary function tests are not used by clinicians.

First, to our knowledge, there has never been a randomized trial of a diagnostic strategy involving routine tests of coronary function linked to medical decisions. Hence, there are no data to confirm that use of the tests in appropriate patients might lead to benefits. Accordingly, the recommendations in practice guidelines (eg, Class IIb) are based on the weakest forms of evidence (Level of Evidence C).^37^ We believe that this lack of evidence reflects the lack of relevant trials and paucity of clinical evidence such that the management of individual patients in daily practice is heterogeneous and empirical. Second, there are some disincentives to the use of these tests, including a modest increase in the duration of the invasive diagnostic procedure and the associated costs. Third, although Ach testing has been shown to be safe and used widely for research purposes,^26^ Ach is not licensed for parenteral use and is not available in daily practice in some health care systems (eg, in the UK National Health Service). Finally, some clinicians may take the view that even if microvascular or vasospastic angina were diagnosed objectively, no specific treatments are available and therapy would be much the same. We suggest that patients and health care providers may take a different view. Clarification of a diagnosis to rule in or rule out a problem will help clinicians to make informed treatment decisions.^49^ Patients with confirmed microvascular or vasospastic angina are content with a precise diagnosis allowing medications to be uptitrated as appropriate. Patients with completely normal invasive assessment of coronary artery function can have antianginal therapy appropriately stopped, and alternative (eg, noncardiac) causes of chest pain can be investigated.

Chest pain is a major cause of planned and unplanned attendances at hospital with potentially avoidable admissions.^11^ Clarification of the diagnosis and treatment may improve patient satisfaction and well-being and help clinicians to appropriately target therapy.^50^ There are potential cost-savings if the enhanced clarification of symptoms translates to future reductions in unnecessary tests, treatments, and hospital attendances for chest pain.

Coronary microvascular dysfunction is associated with poor prognosis.11., 28., 30., 31., 34. Lee et al recently reported data on invasive metrics of coronary artery function in 313 patients with NOCAD (median 658 days). They showed that the group with physiologically important coronary microvascular dysfunction (low CFR and a high IMR) was at the greatest risk of major adverse cardiac events (HR 4.91 [1.54-15.7]; *P* = .007) even compared to multivessel CAD (HR 3.64 [1.24-10.7]; *P* = .019) and diabetes mellitus (HR 2.71 [1.05-7.02]; *P* = .039).^51^ The patients with microvascular angina may benefit from targeted therapy with evidence-based treatments for angina (eg, β-blockers, angiotensin-converting enzyme inhibitors, and statins). Recent position papers call for pathophysiological insights into disease-driving processes that are integrated into a new taxonomy allowing personalized disease management.^52^ The BHF CorMicA trial is the first to assess a routine approach using stratified medicine in patients with angina but nonobstructive coronary disease. This diagnostic intervention is disruptive to current standards of care. Should our hypotheses be confirmed, this developmental clinical trial will inform the design and rationale for undertaking a larger multicenter trial.

## Appendix A Supplementary data

Supplementary File 1. Written management guidance according to endotype. Supplementary File 2. Cardiologist predischarge questionnaire: diagnosis and management. Supplementary File 3. Definition of adverse events. Image 1
