# Supplementary material for: Rationale and design of the British Heart Foundation (BHF) Coronary Microvascular Angina (CorMicA) stratified medicine clinical trial
Source: Am Heart J. 2018 Jul;201:86–94. doi: 10.1016/j.ahj.2018.03.010 (PMC6018570; doi:10.1016/j.ahj.2018.03.010)
Supplement: Supplementary file 1 — Written management guidance according to endotype. Supplementary File 2. Cardiologist predischarge questionnaire: diagnosis and management. Supplementary File 3. Definition of adverse events. [file mmc1.zip › Supp File 1.pdf]

Supplementary File 1. Written management guidance according to endotype

NHS Golden Jubilee National Hospital

Golden Jubilee Research Institute
Patients at the heart of progress

CorMicA Pilot Study- Discharge guidance framework for GPs

Diagnosis – Microvascular angina

We have produced this brief guidance document to assist in managing microvascular angina based on the 2013 ESC guidelines & 2007 SIGN guidelines.[1,2]

Pharmacological management

- Calcium antagonists (e.g. **Verapamil** 40mg **BD** uptitrated weekly according to response)
    - Or Beta-blockers (e.g. **Carvedilol** 6.25mg **BD** uptitrated weekly or to response)
- **Aspirin, Statin** or **ACEI** may be reasonable (depending on patient characteristics)
- Short-acting PRN nitrate (e.g. **Sublingual GTN**)
- **Nicorandil** if refractory symptoms (e.g. 5mg BD uptitrated weekly according to response)
- Xanthine inhibitors (aminophylline) – if refractory to all above

Non Pharmacological lifestyle & risk factor control

- **Smoking** "Smoking is a strong and independent risk factor for CVD and all smoking, including environmental smoking exposure, must be avoided in all patients with CVD"
- **Diet** "A healthy diet reduces CVD risk... Energy intake should be limited to the amount of energy needed to maintain (or obtain) a healthy weight—that is, a BMI <25 kg/m2."
- **Exercise** "moderate-to-vigorous intensity aerobic exercise training ≥3 times a week" (30 min)
- **Weight** "Weight reduction in overweight and obese people is recommended in order to achieve favourable effects on BP, dyslipidaemia and glucose metabolism"
- **Lipids** – "The goals of treatment are LDL-C below 1.8 mmol/L"
- **Hypertension** – "SBP/DBP to values within the range 130–139/80–85 mmHg"
- **Diabetes** "good control of glycated haemoglobin (HbA1c) to <7.0%...based on individual considerations."
- **Psychosocial** "Patients should be assessed for psychosocial distress and appropriate care offered… Refer for psychotherapy, medication or collaborative care in the case of clinically significant symptoms of depression, anxiety and hostility."
- **Cardiac rehabilitation** "A comprehensive risk-reduction regimen, integrated into comprehensive cardiac rehabilitation, is recommended to patients with CAD."

1. Task Force M, Montalescot G, Sechtem U, Achenbach S, Andreotti F, Arden C, et al. 2013 ESC guidelines on the management of stable coronary artery disease: the Task Force on the management of stable coronary artery disease of the European Society of Cardiology. European heart journal. 2013;34(38):2949-3003.
2. SIGN. Guideline No. 96 - Management of stable angina. Edinburgh: Scottish Intercollegiate Guidelines Network (SIGN); 2007.

CorMicA Pilot Study - Discharge guidance framework for GPs

Diagnosis – Vasospastic angina

We have produced this brief guidance document to assist in managing vasospastic angina based on the 2013 ESC guidelines & 2007 SIGN guidelines.[1,2]

Pharmacological management

- Non-dihydropyridine calcium channel blocker (e.g. **Verapamil** initially 40mg BD increasing at weekly intervals as tolerated upto 240-360mg daily)
- +/- **Long-acting nitrates** if symptoms ongoing (scheduled to cover the period of the day in which ischaemic episodes most frequently occur, in order to prevent nitrate tolerance.
- β-Blockers should be avoided.
- **Statin** therapy may be reasonable

Non Pharmacological lifestyle & risk factor control

- **Specific to vasospastic angina** – "exclude cocaine/amphetamine use"
- **Smoking** "Smoking is a strong and independent risk factor for CVD and all smoking, including environmental smoking exposure, must be avoided in all patients with CVD"
- **Diet** "A healthy diet reduces CVD risk... Energy intake should be limited to the amount of energy needed to maintain (or obtain) a healthy weight—that is, a BMI <25 kg/m2."
- **Exercise** "moderate-to-vigorous intensity aerobic exercise training ≥3 times a week" (30 min)
- **Weight** "Weight reduction in overweight and obese people is recommended in order to achieve favourable effects on BP, dyslipidaemia and glucose metabolism"
- **Lipids** – "The goals of treatment are LDL-C below 1.8 mmol/L"
- **Hypertension** – "SBP/DBP to values within the range 130–139/80–85 mmHg"
- **Diabetes** "good control of glycated haemoglobin (HbA1c) to <7.0%...based on individual considerations."
- **Psychosocial** "Patients should be assessed for psychosocial distress and appropriate care offered… Refer for psychotherapy, medication or collaborative care in the case of clinically significant symptoms of depression, anxiety and hostility."
- **Cardiac rehabilitation** "A comprehensive risk-reduction regimen, integrated into comprehensive cardiac rehabilitation, is recommended to patients with CAD."

1. Task Force M, Montalescot G, Sechtem U, Achenbach S, Andreotti F, Arden C, et al. 2013 ESC guidelines on the management of stable coronary artery disease: the Task Force on the management of stable coronary artery disease of the European Society of Cardiology. European heart journal. 2013;34(38):2949-3003.
2. SIGN. Guideline No. 96 - Management of stable angina. Edinburgh: Scottish Intercollegiate Guidelines Network (SIGN); 2007.

Supplementary File 2. Cardiologist Predischarge Questionnaire – Diagnosis & Management

Subject Number………………………..

3 – Post angiogram & Coronary function tests

A - Diagnosis

- *What is the likelihood of* ***coronary heart disease****:*
  No ☐ | Unlikely ☐ | Probable ☐ | Yes☐

- *What is the likelihood of* ***angina due to obstructive coronary heart disease*** *(i.e. >70% stenosis in a main branch or >50% in the left main stem):*
  No ☐ | Unlikely ☐ | Probable ☐ | Yes☐

- *What is the likelihood of* ***angina due to a disorder of coronary function*** *(i.e. microvascular angina or vasospastic angina):*
  No ☐ | Unlikely ☐ | Probable ☐ | Yes☐

- *What is the likelihood of a* ***non-cardiac cause of chest pain****:*
  No ☐ | Unlikely ☐ | Probable ☐ | Yes☐

B - Onward treatment

1. *Will the treatment plan change?*
   Yes ☐ | No ☐
   If yes, in what way(s)?
   Medication ☐ | *PCI* ☐ | *CABG* ☐

2. *Should preventive therapy i.e. anti-platelet & statin therapy, be included?*
   Yes ☐ | No ☐

3. *Should angina therapy be included? (if* ***No skip to section C*** *-* ***Onward investigations****)*
   Yes ☐ | No ☐
   If Yes to 3:
   - *Will you now change the angina therapy?*
     Yes ☐ | No ☐
   - Is the angina treatment intended for a disorder of coronary function e.g. microvascular angina, vasospastic angina?
     Yes ☐ | No ☐

C - Onward investigations

- Do you plan additional diagnostic tests? (if **No skip to section D - Onward management**)
  Yes ☐ | No ☐
  - Is it a cardiovascular test? Echocardiogram, CT scan, MRI, Ambulatory ECG
    Yes ☐ | No ☐
  - Is it a non-cardiovascular test? Ultrasound, CT scan, MRI, endoscopy
    Yes ☐ | No ☐

D - Onward management

- Cardiology follow-up: will you discharge the patient (i.e. no follow-up)?
  Yes ☐ | No ☐
- Other specialty: will you refer the patient to a different speciality e.g. gastroenterology, or suggest to the GP to do so?
  Yes ☐ | No ☐
