# Supplementary material for: Rationale and design of the British Heart Foundation (BHF) Coronary Microvascular Angina (CorMicA) stratified medicine clinical trial
Source: Am Heart J. 2018 Jul;201:86–94. doi: 10.1016/j.ahj.2018.03.010 (PMC6018570; doi:10.1016/j.ahj.2018.03.010)
Supplement: Supplementary file 1 — Written management guidance according to endotype. Supplementary File 2. Cardiologist predischarge questionnaire: diagnosis and management. Supplementary File 3. Definition of adverse events. [file mmc1.zip › Supp File 2 - CorMicA Cardiologist questionnaire.pdf]

NHS Golden Jubilee National Hospital

Subject Number………………………..

Golden Jubilee Research Institute

CorMicA Cardiologist questionnaire – Use in Index Admission

1 - Pre-Angiogram

- *What is your assessment of the patient's symptoms?*

  Typical ☐ | Atypical ☐ | Non-anginal ☐

A – Diagnosis (please indicate likelihood based on all of the available clinical information, including the results of any prior non-invasive tests)

- *What is the likelihood of* ***coronary heart disease****:*

  No ☐ | Unlikely ☐ | Probable ☐ | Yes☐

- *What is the likelihood of* ***angina due to obstructive coronary heart disease*** *(i.e. >70% stenosis in a main branch or >50% in the left main stem):*

  No ☐ | Unlikely ☐ | Probable ☐ | Yes☐

- *What is the likelihood of* ***angina due to a disorder of coronary function*** *(i.e. microvascular angina or vasospastic angina):*

  No ☐ | Unlikely ☐ | Probable ☐ | Yes☐

- *What is the likelihood of a* ***non-cardiac cause of chest pain****:*

  No ☐ | Unlikely ☐ | Probable ☐ | Yes☐

2 – Post angiogram, before randomisation

A - Diagnosis

- *What is the likelihood of* ***coronary heart disease****:*
  No ☐ | Unlikely ☐ | Probable ☐ | Yes☐
- *What is the likelihood of* ***angina due to obstructive coronary heart disease*** *(i.e. >70% stenosis in a main branch or >50% in the left main stem):*
  No ☐ | Unlikely ☐ | Probable ☐ | Yes☐
- *What is the likelihood of* ***angina due to a disorder of coronary function*** *(i.e. microvascular angina or vasospastic angina):*
  No ☐ | Unlikely ☐ | Probable ☐ | Yes☐
- *What is the likelihood of a* ***non-cardiac cause of chest pain****:*
  No ☐ | Unlikely ☐ | Probable ☐ | Yes☐

B - Onward treatment

1. *Will the treatment plan change?*
   Yes ☐ | No ☐
   If yes, in what way(s)?
   Medication ☐ | *PCI* ☐ | *CABG* ☐
2. *Should preventive therapy i.e. anti-platelet & statin therapy, be included?*
   Yes ☐ | No ☐
3. *Should angina therapy be included? (if* ***No skip to section C** - **Onward investigations****)*
   Yes ☐ | No ☐

   If Yes to 3:
   - *Will you now change the angina therapy?*
     Yes ☐ | No ☐
   - Is the angina treatment intended for a disorder of coronary function e.g. microvascular angina, vasospastic angina?
     Yes ☐ | No ☐

C - Onward investigations

- Do you plan additional diagnostic tests? (if **No skip to section D - Onward management**)
  Yes ☐ | No ☐
  - Is it a cardiovascular test? Echocardiogram, CT scan, MRI, Ambulatory ECG
    Yes ☐ | No ☐
  - Is it a non-cardiovascular test? Ultrasound, CT scan, MRI, endoscopy
    Yes ☐ | No ☐

D - Onward management

- Cardiology follow-up: will you discharge the patient (i.e. no follow-up)?
  Yes ☐ | No ☐
- Other specialty: will you refer the patient to a different speciality e.g. gastroenterology, or suggest to the GP to do so?
  Yes ☐ | No ☐

Golden Jubilee National Hospital | Golden Jubilee Research Institute

Subject Number……………………….

3 – Post angiogram & Coronary function tests

A - Diagnosis

- *What is the likelihood of* ***coronary heart disease****:*
  No ☐ | Unlikely ☐ | Probable ☐ | Yes☐

- *What is the likelihood of* ***angina due to obstructive coronary heart disease*** *(i.e. >70% stenosis in a main branch or >50% in the left main stem):*
  No ☐ | Unlikely ☐ | Probable ☐ | Yes☐

- *What is the likelihood of* ***angina due to a disorder of coronary function*** *(i.e. microvascular angina or vasospastic angina):*
  No ☐ | Unlikely ☐ | Probable ☐ | Yes☐

- *What is the likelihood of a* ***non-cardiac cause of chest pain****:*
  No ☐ | Unlikely ☐ | Probable ☐ | Yes☐

B - Onward treatment

1. *Will the treatment plan change?*
   Yes ☐ | No ☐
   If yes, in what way(s)?
   Medication ☐ | *PCI* ☐ | *CABG* ☐

2. *Should preventive therapy i.e. anti-platelet & statin therapy, be included?*
   Yes ☐ | No ☐

3. *Should angina therapy be included? (if* ***No skip to section C*** *-* ***Onward investigations****)*
   Yes ☐ | No ☐

   If Yes to 3:
   - *Will you now change the angina therapy?*
     Yes ☐ | No ☐
   - Is the angina treatment intended for a disorder of coronary function e.g. microvascular angina, vasospastic angina?
     Yes ☐ | No ☐

C - Onward investigations

- Do you plan additional diagnostic tests? (if **No skip to section D - Onward management**)
  Yes ☐ | No ☐
  - Is it a cardiovascular test? Echocardiogram, CT scan, MRI, Ambulatory ECG
    Yes ☐ | No ☐
  - Is it a non-cardiovascular test? Ultrasound, CT scan, MRI, endoscopy
    Yes ☐ | No ☐

D - Onward management

- Cardiology follow-up: will you discharge the patient (i.e. no follow-up)?
  Yes ☐ | No ☐
- Other specialty: will you refer the patient to a different speciality e.g. gastroenterology, or suggest to the GP to do so?
  Yes ☐ | No ☐
