# Supplementary material for: Rationale and design of the British Heart Foundation (BHF) Coronary Microvascular Angina (CorMicA) stratified medicine clinical trial
Source: Am Heart J. 2018 Jul;201:86–94. doi: 10.1016/j.ahj.2018.03.010 (PMC6018570; doi:10.1016/j.ahj.2018.03.010)
Supplement: Supplementary file 1 — Written management guidance according to endotype. Supplementary File 2. Cardiologist predischarge questionnaire: diagnosis and management. Supplementary File 3. Definition of adverse events. [file mmc1.zip › Supp File 3 - CorMicA Definition of adverse events.docx]

# Definition of adverse events

1) Major Adverse Cardiovascular Events (MACE) is the composite of cardiovascular death, non-fatal myocardial infarction, hospitalization for transient ischemic attack or stroke.

2) Major Adverse Cardiac Events are defined as cardiac death, or unplanned hospitalization for MI or heart failure.

PCI and CABG are non-major adverse cardiac events.

3) MI is defined according to the criteria specified in the Third Universal Definition of Myocardial Infarction (including Type 4 MI for PCI and Type 5 for CABG).

4) Contrast-induced nephropathy: is defined as either a greater than 25% increase of serum creatinine or an absolute increase in serum creatinine of 0.5 mg/dL after a radiographic examination using a contrast agent.

Serious adverse events (SAEs), including any that may occur at the time of the index invasive procedure, will be prospectively assessed. All SAEs will be reviewed by an independent clinical event committee that is blind to treatment group allocation.
